# Synthesis of New Polyether Ether Ketone Derivatives with Silver Binding Site and Coordination Compounds of Their Monomers with Different Silver Salts

**DOI:** 10.3390/polym8060208

**Published:** 2016-05-30

**Authors:** Jérôme Girard, Nathalie Joset, Aurélien Crochet, Milène Tan, Anja Holzheu, Priscilla S. Brunetto, Katharina M. Fromm

**Affiliations:** 1 Department of Chemistry, University of Fribourg, Chemin du Musée 9, 1700 Fribourg, Switzerland; jerome.girard@outlook.com (J.G.); nathalie.joset@givaudan.com (N.J.); milene.tan@unifr.ch (M.T.); anja.holzheu@unifr.ch (A.H.); priscilla.brunetto@unifr.ch (P.S.B.); katharina.fromm@unifr.ch (K.M.F); 2 Fribourg Center for Nanomaterials, FriMat, Department of Physics, University of Fribourg, Chemin du Musée 3, 1700 Fribourg, Switzerland; aurelien.crochet@unifr.ch

**Keywords:** PEEK, antimicrobial polymer coating, silver coordination polymer

## Abstract

Polyether ether ketone (**PEEK**) is a well-known polymer used for implants and devices, especially spinal ones. To overcome the biomaterial related infection risks, 4-4′-difluorobenzophenone, the famous **PEEK** monomer, was modified in order to introduce binding sites for silver ions, which are well known for their antimicrobial activity. The complexation of these new monomers with different silver salts was studied. Crystal structures of different intermediates were obtained with a linear coordination between two pyridine groups and the silver ions in all cases. The mechanical and thermal properties of different new polymers were characterized. The synthesized **PEEKN5** polymers showed similar properties than the **PEEK** ones whereas the **PEEKN7** polymers showed similar thermal properties but the mechanical properties are not as good as the ones of **PEEK**. To improve these properties, these polymers were complexed with silver nitrate in order to “cross-link” with silver ions. The presence of ionic silver in the polymer was then confirmed by thermogravimetric analysis (TGA) and X-ray powder diffraction (XRPD). Finally, a silver-based antimicrobial compound was successfully coated on the surface of **PEEKN5**.

## 1. Introduction

Benzophenone is a widely used organic compound, for example, as a photo-initiator [1] in inks [2] and imaging. One of its derivatives is used as a monomer for the preparation of polyether ether ketone (**PEEK**) [3], which is a thermoplastic polymer with good mechanical and chemical properties [4,5]. **PEEK** is used in many different applications, such as cable insulation, pumps, and also in medicine, in particular for spine implants [6]. Polymer implants must be fatigue resistant, biocompatible, and resistant in aqueous media [7] and they should maintain these properties for many years. One of the main problems for body-foreign (e.g., polymers, metals) implants is the infection rate, which is overall 5% [8], and mostly due to bacterial adhesion and biofilm formation [9]. A possible solution to decrease the infection rate is to cover the implant surface with coatings having antimicrobial and antifungal properties. Antibiotics have been used in this context, but they are prone to degradation and may give rise to bacterial resistance [10]. Another promising type of coating that our group has been involved with is based on silver ion coordination compounds/polymers [11,12].

The silver ion toxicity against bacteria is well known and is still used in, e.g., cosmetics or creams for burn wounds [13,14]. Silver-containing coatings have already been attached to the surface of metallic implants by surface modification and showed good antimicrobial properties and biocompatibility [15,16,17]. However, for polymer-based implant pieces, surface modification is quite challenging without degrading the properties of the material [18]. One solution is to modify the polymer by covalently introducing binding sites for silver ions (electron donor systems like nitrogen or oxygen atoms) in order to grow antibacterial coatings on the implant surface but also to have silver inside the polymer for slow silver release.

**Scheme 1:**
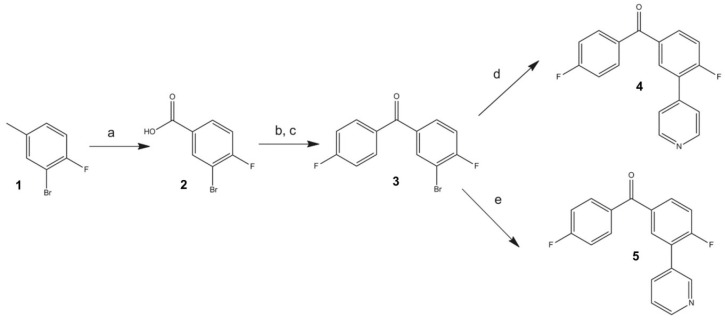
Reaction pathway for **4** and **5**: (**a**) Na_2_Cr_2_O_7_, H_2_SO_4_; (**b**) oxalyl chloride, CH_2_Cl_2_; (**c**) LiCl, AlCl_3_, dichloroethane, fluorobenzene; (**d**) 4-pyridinylboronic acid, K_2_CO_3_, Pd(PPh_3_)_4_, DMF, H_2_O; (**e**) 3-pyridinylboronic acid, K_2_CO_3_, Pd(PPh_3_)_4_, DMF, H_2_O.

In this work, new derivatives of **PEEK** monomers are presented, and their binding to silver ions is investigated. Crystal structures of silver complexes of monomeric moieties are studied: 3-pyridine-4-yl-4,4′-difluorobenzophenone (**4**) and 3-pyridine-3-yl-4,4′-difluorobenzophenone (**5**) with a pyridyl group for the silver binding and two fluorine atoms for polymerization. They were synthesized by first complete oxidation of 3-bromo-4-fluorotoluene [19], then chlorination of the acid [20] followed by a Friedel-Crafts reaction between the acyl chloride and fluorobenzene [21], and, finally, a Suzuki coupling with the corresponding boronic acid (Scheme 1). Intermediates and silver complexes of the final products were characterized by single crystal X-ray crystallographic analyses.

## 2. Materials and Methods

### 2.1. Materials

All chemicals were standard reagent grades and were used without further purification. Air-sensitive and/or moisture-sensitive reactions were conducted under a dry argon atmosphere. ^1^H, ^13^C, ^14^F and ^31^P-NMR spectra were recorded with 300 or 500 MHz spectrometers with residual solvent signals used as a reference. All the reactions subjected to microwave heating were performed in a Biotage^®^ Initiator 2.0 (Biotage, Uppsala, Sweden), 400 W microwave. Column chromatography was carried out on silica gel. DSC analyses were done on a Mettler-Toledo DSC 30 (Mettler-Toledo, Bussigny, Switzerland) with a TC 15 controller (Mettler-Toledo, Bussigny, Switzerland) and TGA analyses were done on a TGA/SDTA 851e (Mettler-Toledo, Bussigny, Switzerland). All crystals were mounted on loops and all geometric and intensity data were taken from one single crystal. Data collection was performed either using Mo-Kα radiation (λ = 0.71073 Å) at 200 K on a STOE IPDS-II diffractometer, or using Cu-Kα radiation (λ = 1.54186 Å) at 200 K on a STOE IPDS-IIT diffractometer (STOE, Darmstadt, Germany), both machines are equipped with an Oxford Cryosystems open flow cryostat [22]. Absorption correction was partially integrated in the data reduction procedure [23]. The structure was solved by SIR 2004 [24] or SHELX-97 and refined using full-matrix least-squares on *F*^2^ with the SHELX-97 package [24,25]. All heavy atoms could be refined anisotropically. Hydrogen atoms were introduced as fixed contributors when a residual electronic density was observed near their expected positions. The mechanical properties of the polymers were characterized by dynamic mechanical thermal analysis (DMTA, TA Instruments, TA Instruments, New Castle, DE, USA, Model Q800). Tests were conducted in tensile mode using a temperature sweep method (50–250 °C) at a fixed frequency of 1 Hz.

Crystallographic data have been deposited with the Cambridge Crystallographic Data Centre, CCDC, 12 Union Road, Cambridge CB21EZ, UK. Copies of the data can be obtained on quoting the depository numbers CCDC-1411840 to -1411845 for compound **2**, **3**, **4**, **4a**, **4b** and **5a**, respectively. (http://www.ccdc.cam.ac.uk/conts/retrieving.html).

### 2.2. Preparation of 3-Bromo-4-Fluorobenzoic Acid (**2**)

In a round-bottomed flask, fitted with a stirrer, 53.2 g (0.179 mol) of sodium dichromate, 120 mL of water, and 25 g (0.132 mol) of 3-bromo-4-fluorotoluene (**1**) were placed. Stirring was started, and 130 g of concentrated sulfuric acid was added. After the spontaneous heating of the reaction mixture had subsided, the mixture was heated to gentle boiling for 12 h. Two hundred milliliters of water was added to the solution, cooled down and filtered out. The product was washed with 100 mL of water. Next, the crude product was warmed and stirred with 100 mL of dilute sulfuric acid solution. After cooling, the product was again filtered out and then dissolved in dilute sodium hydroxide solution and filtered out. The filtrate, which should be light yellow or greenish in color, was acidified with dilute sulfuric acid while stirring. The precipitated product was filtered, washed thoroughly with water, and dried to give product (**2**): 7.8 g (27%). ^1^H-NMR (300 MHz, CD_2_Cl_2_) δ 7.27–7.22 (t, *J* = 8.3 Hz, 1H) 8.10–8.05 (ddd, *J* = 2.2, 4.6, 8.3 Hz, 1H) 8.36–8.33 (dd, *J* = 2.2, 6.5 Hz, 1H). ^13^C-NMR (75 MHz, CD_2_Cl_2_) δ 110, 117, 127, 132, 136, 161, 165. IR *ν* = 3054 (mb), 2811 (mb), 2542 (mb), 1928 (w), 1805 (w), 1675 (s), 1589 (s), 1491 (m), 1422 (s), 1262 (s), 1047 (m), 764 (s), 682 (s), 625 (s). MS (ESI): *m*/*z* 126.93, 128.93 [(M − H)^−^, calcd 126.93, 128.93].

### 2.3. Preparation of 3-Bromo-4,4′-Difluorobenzophenone (**3**)

To a solution of (**2**) (11 g, 50 mmol) in dry dichloromethane (100 mL) at 0 °C under an argon atmosphere, oxalyl chloride (8.8 mL, 92 mmol) was dropwise added, followed by a few drops of dimethylformamide. The ice bath was removed, the reaction was heated to reflux for 12 h, and then cooled down. Afterwards, the solvent was removed under vacuum to afford a light-brown oil. It was placed under reduced pressure to remove residual oxalyl chloride. A mixture of the acyl chloride (11.87 g, 0.05 mol) and fluorobenzene (4.8 g, 0.05 mol) in dichloroethane (7 mL) was dropwise added to a stirred mixture of lithium chloride (3.18 g, 0.075 mol) and aluminium chloride (20 g, 0.15 mol) in dichloroethane (20 mL) at −15 °C. The reaction mixture was removed from the cooling bath after one hour, mixed at 0 °C for three hours and then stirred at room temperature overnight. The reaction mixture was now slowly added to 100 mL of a stirred mixture of ice and dilute aqueous hydrochloric acid, at which time the organic phases separated. The aqueous phase was washed twice with 50 mL of diethylether. Finally the combined organic phases were washed with 50 mL of diluted sodium hydroxide solution, then water, dried and evaporated to give product (**3**): 14.1 g (95%) ^1^H-NMR (300 MHz, CD_2_Cl_2_) δ 7.29–7.17 (m, 3H) 7.75–7.70 (ddd, *J* = 2.2, 4.7, 8.5 Hz, 1H) 7.82–7.78 (dd, *J* = 5.4, 8.8 Hz, 2H) 8.03–8.00 (dd, *J* = 2.2, 6.7 Hz, 1H). ^13^C-NMR (75 MHz, CD_2_Cl_2_) δ 110, 117, 132, 133, 134, 136, 161, 166, 193. IR *ν* = 3070 (w), 1924 (w), 1794 (w), 1649 (s), 1588 (s), 1486 (m), 1390 (m), 1261 (s), 1153 (s), 1046 (m), 959 (m), 904 (m), 851 (s), 759 (s), 673 (s), 583 (s). MS (MALDI): *m*/*z* 296.97, 298.97 [(M + H)^+^, calcd 296.97, 298.97].

### 2.4. Preparation of 3-Pyridin-4-yl-4,4′-Difluorobenzophenone (**4**)

Compound (**3**) (25 mg, 0.084 mmol), 4-pyridylboronic acid (15.5 mg, 0.126 mmol), tetrakis(triphenylphosphine)palladium(0) (10 mg, 0.008 mmol) and potassium carbonate (50 mg, 0.36 mmol) in dimethylformamide (3.5 mL) and water (0.5 mL) were mixed together under inert conditions. The reaction was heated to 160 °C for 10 min using microwave irradiation. The reaction solution was concentrated to dryness and the residue was taken up in water and was two times extracted with ethyl acetate. Then, the organic extracts were combined, dried over magnesium sulfate and evaporated. Finally, the crude product was purified by silica gel chromatography (ethyl acetate/hexane (1/1), then pure ethyl acetate) to give compound (**4**): 18 mg (73%). ^1^H-NMR (300 MHz, CD_2_Cl_2_) δ 7.25–7.17 (m, 2H) 7.36–7.30 (dd, *J* = 8.6, 10.2 Hz, 1H) 7.52–7.49 (dd, *J* = 1.6, 4.4 Hz, 2H) 7.88–7.81 (m, 3H) 7.96–7.93 (dd, *J* = 2.2, 7.3 Hz, 1H) 8.69–8.67 (dd, *J* = 1.6, 4.4 Hz, 2H). ^13^C-NMR (75 MHz, CD_2_Cl_2_): δ 116, 117, 124, 127.1, 127.3, 133, 134, 135, 142, 151, 161, 164, 168. IR *ν* = 3057 (w), 1653 (s), 1590 (s), 1502 (m), 1400 (m), 1306 (m), 1223 (s), 1129 (m), 1042 (w), 993 (w), 858 (m), 763 (s), 694 (m), 585 (s). MS(ESI): *m*/*z* 296.09 [(M + H)^+^, calcd 296.09].

### 2.5. Preparation of 4-Pyridin-4-yl-4,4′-Difluorobenzophenone (**5**)

Compound (**3**) (100.1 mg, 0.337 mmol), 3-pyridylboronic acid (62.1 mg, 0.505 mmol), tetrakis(triphenylphosphine)palladium(0) (40.4 mg, 0.035 mmol) and potassium carbonate (203.6 mg, 1.473 mmol) were mixed together in dimethylformamide (14 mL) and water (2 mL) under inert conditions. The reaction mixture was heated to 160 °C for 10 min using microwave irradiation. Afterwards, the resulting reaction mixture was concentrated to dryness and the residue was taken up in water and was two times extracted with ethyl acetate. The organic extracts were combined and dried over magnesium sulfate and evaporated. Finally, the crude product was purified by silica gel chromatography (ethyl acetate/hexane (3/1) then pure ethyl acetate). The expected product (**5**) was a light yellow oil, 77 mg (0.26 mmol, 72%). ^1^H-NMR (300 MHz, CDCl_3_): δ 7.13–7.25 (m, 2H), 7.31 (dd, *J* = 9.82, 8.5 Hz, 1H), 7.41 (ddd, *J* = 7.98, 4.86, 0.76 Hz, 1H), 7.77–7.98 (m, 5H), 8.65 (dd, *J* = 4.82, 1.42 Hz, 1H) 8.82 (s, 1H); ^13^C-NMR (75 MHz, CDCl_3_): δ 115, 116, 123, 126, 130, 132.0, 132.5, 132.6, 133, 134, 136, 149, 150, 162, 166, 193. IR: ν = 3062 (w), 3038 (w), 1735 (w), 1657 (s), 1595 (s), 1503 (m), 1416 (m), 1317 (m), 1260 (s), 1225 (s), 1156 (s), 1122 (m), 1020 (w), 942 (m), 851 (m), 765 (s), 711 (s). MS(ESI): *m*/*z* 296.09 [(M + H)^+^, calcd 296.09].

### 2.6. Preparation of 3-Pyridin-4-yl-4,4′-Difluorobenzophenone Silver Trifluoromethanesulfonate Complex (**4a**)

Compound (**4**) (50 mg, 0.17 mmol) was mixed with silver trifluoromethanesulfonate (22 mg, 0.085 mmol) in dichloromethane. Slow evaporation of the mixture gave colorless plates of complex (**4a**) suitable for X-ray measurement. ^1^H-NMR (300 MHz, CD_2_Cl_2_): δ 7.25–7.19 (m, 2H), 7.41–7.35 (dd, *J* = 8.6, 10.2 Hz, 1H), 7.78–7.76 (m, 2H), 7.93–7.82 (m, 4H), 8.67–8.65 (dd, *J* = 1.6, 4.4 Hz, 2H). IR: ν = 3100 (w), 2923 (w), 1658 (m), 1597 (m), 1485 (w), 1432 (w), 1223 (s), 1155 (s), 1024 (s), 958 (m), 851 (m), 763 (s), 694 (w), 634 (s), 585 (s). Calcd. C 52.4, H 2.62, N 3.31, S 3.78; Found C 53.1, H 2.66, N 2.97, S 3.99.

### 2.7. Preparation of 3-Pyridin-4-yl-4,4′-Difluorobenzophenone Silver Methanoate Complex (**4b**)

Compound (**4**) (50 mg, 0.17 mmol) was mixed with silver nitrate (14.5 mg, 0.085 mmol) in methanol/THF 1:1 mixture. Slow evaporation gave white needles of complex (**4b**) suitable for X-ray measurement. IR: ν = 3420 (w), 3071 (w), 1658 (m), 1597 (m), 1485 (w), 1396 (m), 1294 (s), 1223 (s), 1155 (s), 1024 (s), 958 (m), 851 (m), 763 (s), 694 (w), 634(s), 585 (s).

### 2.8. Preparation of 3-Pyridin-3-yl-4-4′-Difluorobenzophenone Silver Nitrate Complex (**5a**)

Compound (**5**) (51 mg, 0.17 mmol) dissolved in 3 mL of dichloromethane was mixed with silver nitrate (15.9 mg, 0.094 mmol) dissolved in 3 mL of methanol. Slow evaporation of the mixture gives white needles of complex (**5a**) suitable for X-ray measurement. ^1^H-NMR (300 MHz, CDCl_3_): δ 7.17–7.26 (m, 2H), 7.33 (dd, *J* = 9.91, 8.59 Hz, 1H), 7.56 (dd, *J* = 7.55, 5.29 Hz, 1H), 7.78–7.96 (m, 4H), 8.04 (d, *J* = 7.18 Hz, 1H) 8.73 (s, 1H), 8.89 (s, 1H); ^13^C-NMR (75 MHz, CDCl_3_): δ 115.8, 116.6, 124.7, 132.6, 133.2, 134.5, 138.1, 150.7, 151, 162.1, 165.6, 193; IR: ν = 3070 (w), 1656 (m), 1595 (s), 1503 (m), 1427 (w), 1379 (w), 1317 (s), 1230 (s), 1156 (m), 1126 (m), 1042 (w), 945 (m), 853 (m), 812 (w), 767 (m), 754 (m), 733 (m), 701 (m).

### 2.9. General Procedure for Polymerization of Polyether-Ether-Ketone-3-Pyridiny-4-yl (**PEEKNx** = **PEEKN5**, **PEEKN6** and **PEEKN7**)

**Scheme 2:**
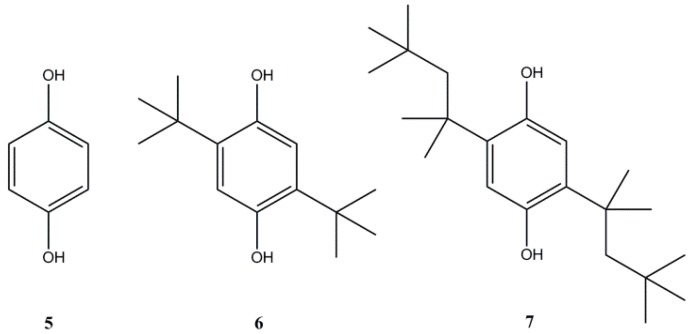
Different molecules used as monomers for the polymerization.

In a 2-necked flask equipped with a stirrer, air condenser and argon inlet, product (**4**) and hydroquinone derivatives (**5** for **PEEKN5**, **6** for **PEEKN6** or **7** for **PEEKN7**) (Scheme 2) were added in a 1:1 ratio with diphenylsulphone as solvent and potassium carbonate (1.05 equiv.). The mixture was heated to 200 °C for 30 min, then to 250 °C for 30 min, and finally to 300 °C for 30 min. After cooling, the resulting product was ball milled and washed with water, acetone and methanol or only water and methanol in case of **PEEKN7**. For **PEEKN5** IR: ν = 3041 (w), 1653 (m), 1594 (m), 1491 (s), 1415 (w), 1314 (s), 1218 (s), 1185 (s), 946 (w), 825 (m), 763 (m), 698 (w). Calcd. C 78.9, H 4.14, N 3.83; Found C 78.2, H 4.05, N 3.57. For **PEEKN7** ^1^H-NMR (300 MHz, CDCl_3_): δ 0.78–0.60 (18H) 1.30–1.20 (12H) 1.67 (4H) 7.20–6.90 (5H) 7.57 (2H) 7.95–7.79 (4H) 8.68 (2H). IR: ν = 2952 (m), 2870 (w), 1658 (m), 1597 (m), 1477 (m), 1391 (m), 1365 (m), 1315 (m), 1216 (s), 1161 (s), 944 (w), 848 (w), 762 (m), 698 (w) Calcd. C 81.5, H 8.03, N 2.37; Found C 80.9, H 7.50, N 2.37.

### 2.10. Coating of **PEEKNx** with Ethanediyl Bis(isoniconate) Silver Nitrate Complex

Pieces of **PEEKNx** were placed into a mixture solution containing ethanediyl bis(isoniconate) and silver nitrate at a 4 mM concentration dissolved in THF/EtOH (1/1). After a 24-h incubation time, our test specimens were rinsed three times with pure ethanol.

### 2.11. Antimicrobial Assay

Kirby-Bauer assays were performed with the insoluble **PEEKN5** polymer coated with ethanediyl bis(isoniconate) silver nitrate complex. In addition, 10 µL of 0.6, 0.15, 0.0375, 9.375 × 10^−3^, 2.34 × 10^−3^ and 5.85 × 10^−4^ mol·L^−1^ AgNO_3_ solutions in Milli-Q water deposited on 1 cm^2^ Whatman filter papers grade 1 were also tested. Both type of samples were prepared in triplicates, embedded into Mueller Hinton agar II (Conda, cat. 1055.00) and incubated for 3 h at 37 °C. 50 µL of the 1 × 10^6^ CFU·mL^−1^ *Escherichia coli* (*E. coli*) suspension (ATCC^®^25922) in Mueller Hinton Broth media (Sigma Aldrich, cat. 70192, St. Louis, MO, USA) was streaked on top. On the next day the inhibition zones are measured with a ruler (6 measurement points were taken from each sample). Agar and *E. coli* suspension were used as positive and negatives controls, respectively.

## 3. Results and Discussion

### 3.1. X-Ray Diffraction

Since intermediate products for the synthesis of **PEEKNx** gave single crystals with interesting structural features (Table S1), we wish to present their structures briefly in the following.

**Figure 1:**
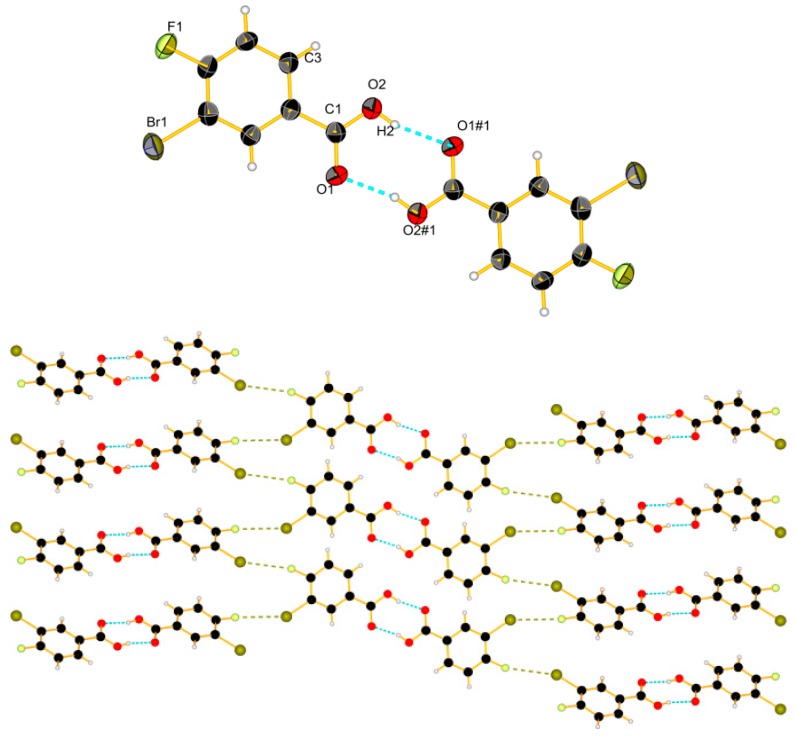
View of the molecular structure of **2**: (**top**) with hydrogen bonds in blue dashed lines, 50% probability (#1 = −*x*, 2 − *y*, −*z*); (**bottom**) packing with short contacts between halogen atoms in khaki dashes.

Product **2** was dissolved in chloroform, and a slow evaporation of the solvent lead product **2** to form colorless needles in the monoclinic space group *P*2_1_/n. In the solid state, the molecules form dimers by hydrogen bonding $R_{2}^{2}\left(8\right)$ between two acid functions [26,27] C–OH2∙∙∙O1#1 (1.807(5) Å) (Figure 1). There are also intermolecular F1∙∙∙Br1 short contacts (3.177(5) Å), leading to a packing of the molecules into layers of dimers, with each layer being linked by halogen short contacts to neighbour pairs and with a twist of *ca.* 53° from one pair to the next.

**Figure 2:**
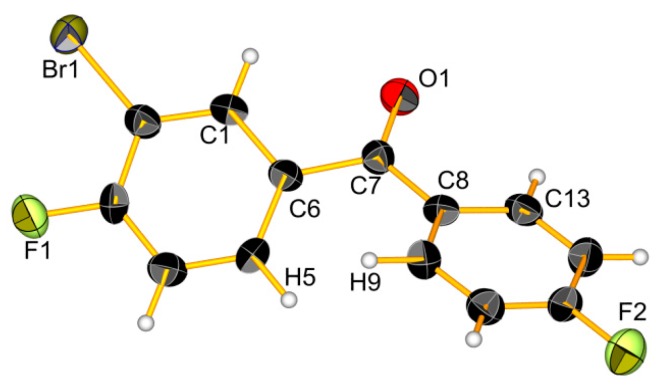
View of the molecular structure of **3** in ellipsoid 50% probability.

Product **3** crystallized, as **2**, in the monoclinic space group *P*2_1_/c (Figure 2). Conjugation of the carbonyl group with the two phenyl rings would favour a planar conformation [28] but a steric repulsion between H9 and H5 induces a torsion angle between the trisubstituted ring and the carbonyl group of 24.6(9)° and of 21.7(9)° between the disubstituted ring and the carbonyl group. For comparison, the torsion angle in the 4,4′-difluorobenzophenone [29] is 23.9(5)°. The packing (Figure S1) is formed by layers of molecules; each molecule is connected to two others via halogen short contacts, similar to the crystal structure of **2**. The distance between F2∙∙∙Br1 is 3.123(4) Å.

**Figure 3:**
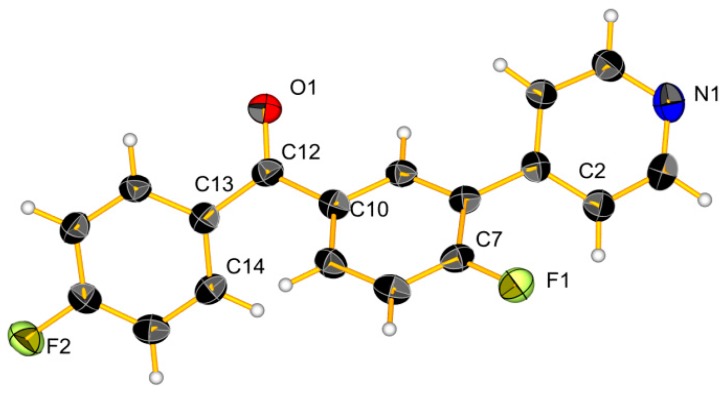
View of the molecular structure of **4** in ellipsoid 50% probability.

Product **4** crystallized by slow evaporation from dichloromethane to colorless blocks, in the monoclinic space group *P*2_1_/c (Figure 3). The torsion angles are larger than for **3**, respectively 26.2(5)° and 29.6(5)°, the one between the pyridine and the phenyl ring is 34.0(4)°. The molecules of **4** pack (Figure S2) in waves, and π–π stacking is observable for the pyridine ring with a distance of *ca.* 3.78 Å.

**Figure 4:**
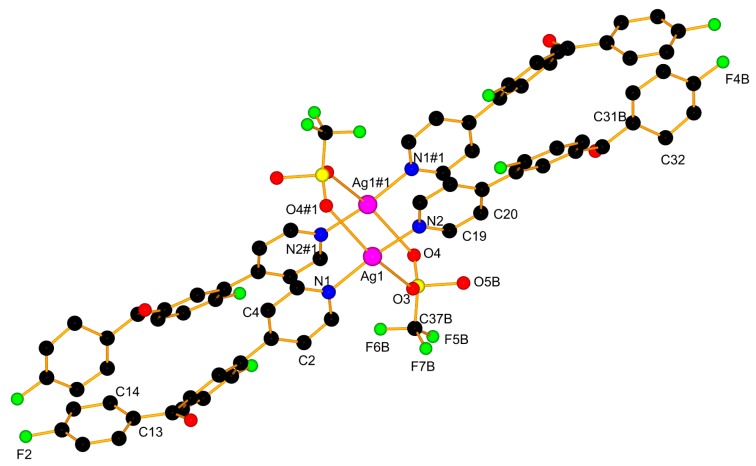
View of the dimer structure of complex **4a**, hydrogen atoms are omitted for clarity (#1 = 0.5 + *x*, −0.5 − *y*, −0.5 + *z*).

When product **4** was mixed with silver trifluoromethanesulfonate a complex formed, which crystallized in the monoclinic space group *C*2/c to form compound (**4a**). Each silver ion is coordinated by two pyridine moieties with a distance of 2.136(7) Å for Ag1–N1 and 2.139(7) Å for Ag1–N2, and with an angle N1–Ag1–N2 of 172.1(2). Two silver ions are pairwise bridged by two different trifluoromethanesulfonate anions, which bind via two oxygen atoms each. The Ag–Ag distance is 3.2578(8) Å (Figure 4), which is too long for a metal-metal distance. In **4a**, disorder is observed for the counter ion, in which the trifluoromethane part is oriented 50% to the left and the other half to the right with respect to the Ag‒Ag vector. The phenyl ring constituted by the carbon atoms C31 to C36, except C32 and F4, is also disordered and split into two different positions of 0.5 occupancy. Structure resolution was tried with a larger unit cell in order to find a superstructure but without success. We can conclude that the position of the phenyl ring and the counter ion are purely statistical.

Torsion angles for the first ligand containing N1 are 16(1)° between the trisubstituted phenyl and the carbonyl group, 44(1)° between the disubstituted phenyl and the carbonyl group, and 41(1)° between the pyridine and the phenyl rings. For the second ligand containing N2 the torsion angles are 38(1)° or 17(1)° for the triubstituted phenyl and the carbon (the second value of the torsion angle is due to the disorder of the phenyl ring), 13(2)° or 30(2)° for the disubstituted phenyl and the carbonyl group (depending on the disorder position) and 29(1)° between the pyridine and the phenyl rings. Thus, the coordination and packing effects seem to influence these angles quite strongly.

**Figure 5:**
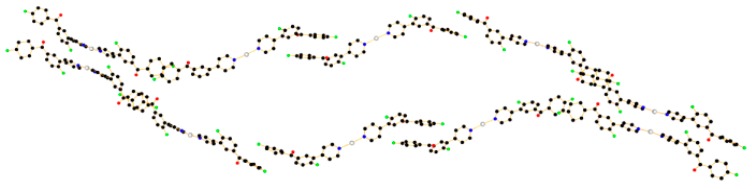
View of the packing of **4a**. Hydrogen Atoms and trifluoromethane sulfonate were omitted for clarity. Different colors have been attributed to the different atoms (black: C, blue: N, red: O, yellow: P, green: F, grey: Ag).

**Figure 6:**
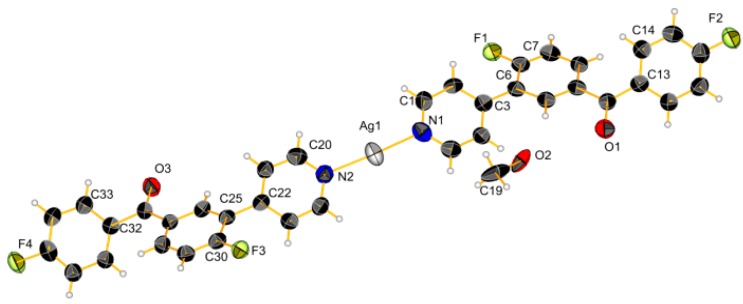
View of the complex **4b** in ellipsoid 50% probability.

Packing of **4a** (Figure 5) has the shape of a wave, each silver atom is bound to two ligands, the disubstituted ring of one ligand shows weak π–π interactions with the disubstituted ring of the neighbour ligand (distance around 4.0 Å). On the other side, the second ligand features a π–F interaction [30] at a distance of 3.5 or 3.8 Å (the second value is due to the disorder in the ring). These two interactions lead to a polymer chain, and each five silver atoms, the same polymer chains “meet” to form the dimer via the bridging anions. The silver ions in between connect to other similar motifs in the structure, so that each chain is thus linked to many other chains by the dimer formation by silver atom pairs. Compound **4** also complexes with silver methanoate and crystallized into fine needles of monoclinic space group *P*2_1_/c (Figure 6).

**Figure 7:**
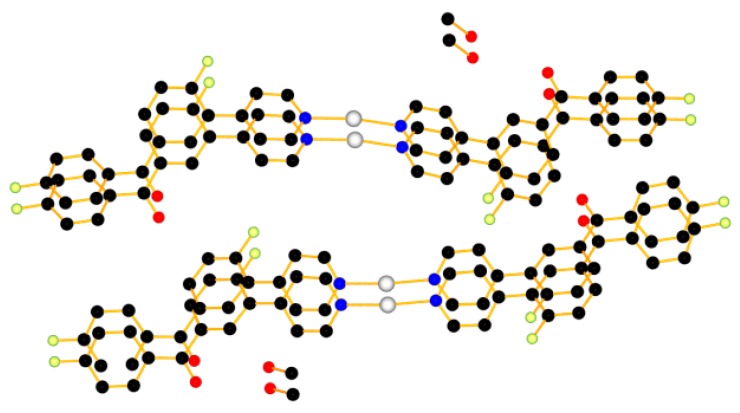
View of the packing of **4b**. Hydrogen atoms were omitted for clarity. Different colors have been attributed to the different atoms (black: C, blue: N, red: O, yellow: P, green: F, grey: Ag).

Each silver atom is coordinated by two pyridine moieties with a distance of 2.156(7) Å for Ag1–N1 and 2.154(7) Å for Ag1–N2 and an angle of 171.3(3)°. The distance between two silver atoms of parallel complexes is 3.826(1) Å. The torsion angles are different from the previous complex **4a**, *i.e.*, the torsion angle between the trisubstituted phenyl and the carbonyl group is 23(1)°, 30(1)° for the one between the disubstituted phenyl and the carbonyl group, and 30(1)° for the one between the pyridine ring and the phenyl group. The packing of **4b** is governed by π–π stacking. Each ring of a ligand molecule interacts via π–π stacking with another cycle with a distance of 3.8 Å (Figure 7). The difference between the torsion angles of the two complexes can be explained by the difference of packing in the two crystal structures and the different role of the anions, which do not act as bridging ligands.

Interestingly, the starting salt for the formation of **4b** was silver nitrate, but in the structure the counter ion is methanolate. Indeed, the methanol, which was used as solvent in a mixture with THF, was deprotonated. In this crystal structure, one methanol molecule per asymmetric unit in addition to the anion is completely disordered and cannot be described by the X-ray structure. Its presence is proven by TGA analysis (Figure S3), which shows a weight loss of 4.7% between 50 and 120 °C (for comparison, a methanol molecule represents 4.2%). The NMR spectrum of the product presents also two peaks from 3.2 to 3.6 ppm which likely correspond to the methanol and the methanolate proton.

**Figure 8:**
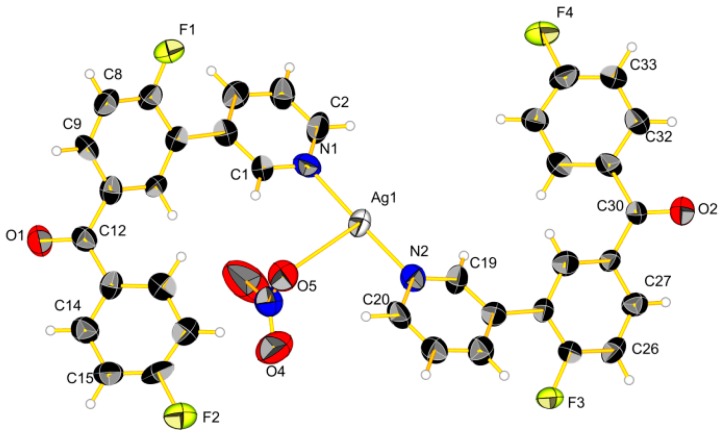
View of the complex **5a** in ellipsoid 50% probability.

When product **5** was mixed with silver nitrate a complex formed, which crystallized in the monoclinic space group *P*2_1_/n to form compound (**5a**) (Figure 8).

**Figure 9:**
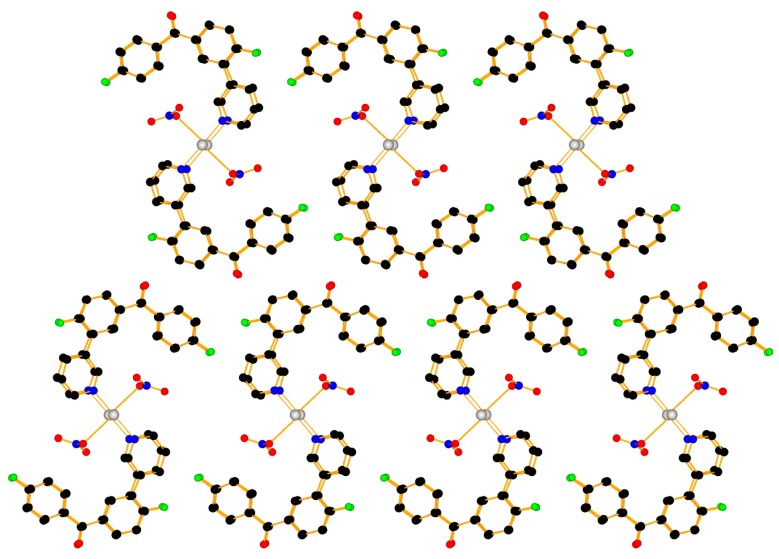
View of the packing of **5a**. Hydrogen atoms were omitted for clarity. Different colors have been attributed to the different atoms (black: C, blue: N, red: O, yellow: P, green: F, grey: Ag).

Each silver atom is coordinated by two pyridine moieties and one oxygen of the nitrate with a distance of 2.150(9) Å for Ag1–N1, 2.133(9) Å for Ag1–N2, and 2.78(1) Å for Ag1–O5 with an angle of 176.6(3)° for N1–Ag1–N2. . The torsion angle between the trisubstituted phenyl and the carbonyl group is 37(1)°, 7(1)° for the one between the disubstituted phenyl and the carbonyl group, and 36(1)° for the one between the pyridine ring and the phenyl group for the molecule containing N1 and 34(1)°, 8(1)° and 39(1)° for the one containing N2, respectively. The methanol molecule in the crystal structure is completely disordered. Indeed one methanol molecule per asymmetric unit is completely disordered and cannot be described by the X-ray structure. An empty channel can be seen in the packing of **5a** (Figure 9) where methanol molecules should be present.

### 3.2. Polymerisation

Product **4** was used to carry out a step-growth polymerization reaction in a polar aprotic solvent, diphenylsulphone, with a high boiling temperature. The final polymer **PEEKN5** was obtained by polymerization of **4** with hydroquinone **5** to give an amorphous brown polymer which melts around 230 °C and decomposes at *ca*. 380 °C (Table 1). The polymer is only soluble in concentrated sulphuric acid, like **PEEK**. This might be due to the high aromaticity of the polymer, which can lead to multiple π–π stackings. In order to improve the solubility, the use of derivatives of hydroquinone, namely **6** and **7**, with side groups that have a big steric effect in order to break the π–π stacking seemed to be appropriate (Scheme 2).

**Figure 10:**
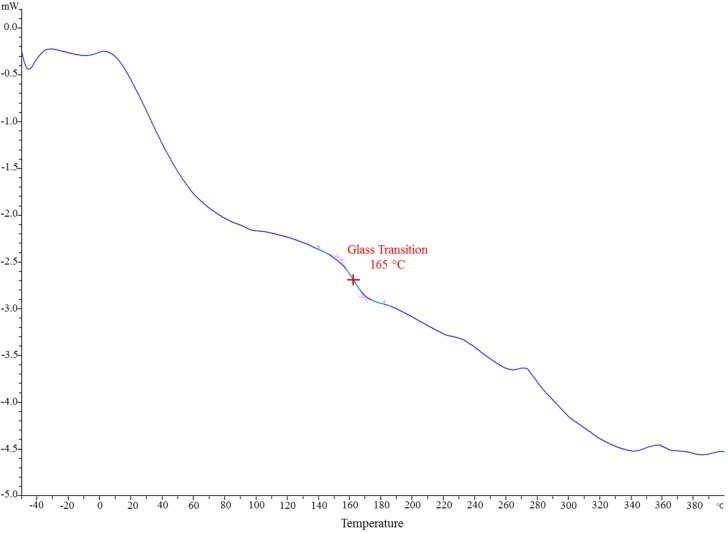
DSC analysis of PEEKN7.

The first test was made with the 2,5-di-tert-butylhydroquinone (**6**). The solubility of the obtained polymer was better in organic solvents like tetrahydrofurane and acetone, but still not good. Thus, we decided to take a larger side group like 2,5-bis(1,1,3,3-tetramethylbutyl)hydroquinone (**7**), whose polymerization with **4** gives a light brown solid (**PEEKN7**) which is soluble in acetone, tetrahydrofurane, dichloromethane, *etc*. The DSC analysis (Figure 10) gives a glass transition temperature of 165 °C. The melting temperatures (Tm) of the synthesized polymers are around 230 °C for **PEEKN5** and 270 °C for **PEEKN7**, whereas the melting temperature of **PEEK** is typically around 340 °C (Table 1). The difference could be due to the introduction of the sterically demanding pyridine rings, which introduce gaps in the structure. The difference of melting temperature between **PEEKN5** and **PEEKN7** could be due to the stronger London interactions in **PEEKN7** with respect to **PEEKN5** [31,32].

**Figure 11:**
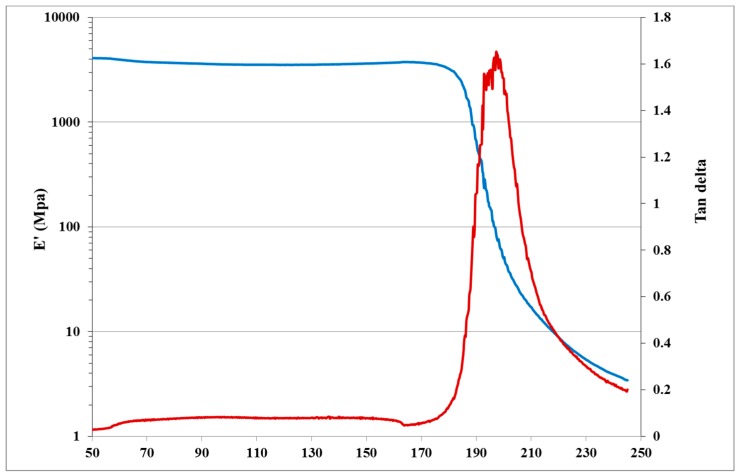
Evolution of the storage modulus (red curve) and tan δ (blue curve) as a function of time for **PEEKN5**.

Thin films (~0.05 mm) of **PEEKN5** and **PEEKN7** were prepared by using a hot press in order to perform a Dynamic Mechanical Thermal Analysis (DMTA) to assess the viscoelastic behavior of polymers. Generally a stress is applied to the material and the deformation is measured. With this deformation we can obtain the dynamic modulus of the material at different temperatures giving the access to the determination of glass transition temperature [33]. Thin films of **PEEKN7** were too brittle to be measured in DMTA, whereas, for **PEEKN5** (Figure 11) tan δ shows a peak at 190 °C which corresponds to the glass transition of the polymer. *T*_g_ of **PEEKN5** is around 50 °C higher compared to **PEEK** [4]. The storage modulus of approximately 4 GPa at 50 °C which is in the same range as commercial **PEEK**, and E′ is stable until 180 °C after which the modulus decreases until 3 MPa at 245 °C (Table 1).

### 3.3. Coating of **PEEKN** with Silver Species

**Figure 12:**
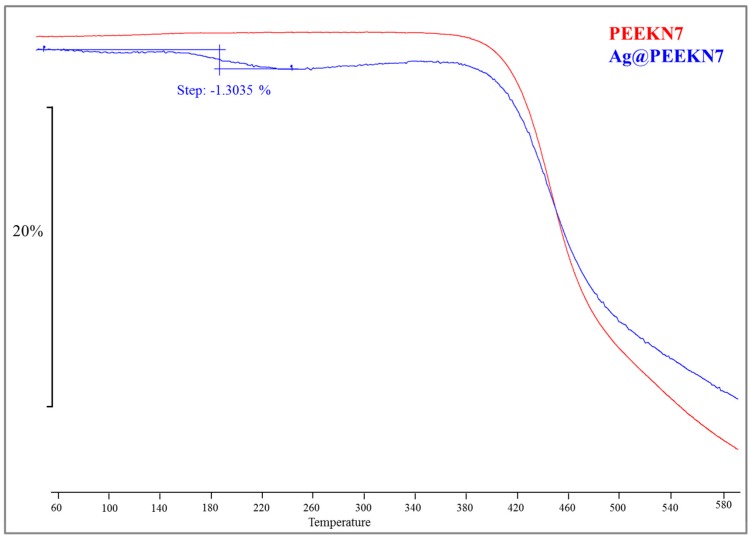
Thermogravimetry analysis of the precipitate of silver nitrate with **PEEKN7**.

**Figure 13:**
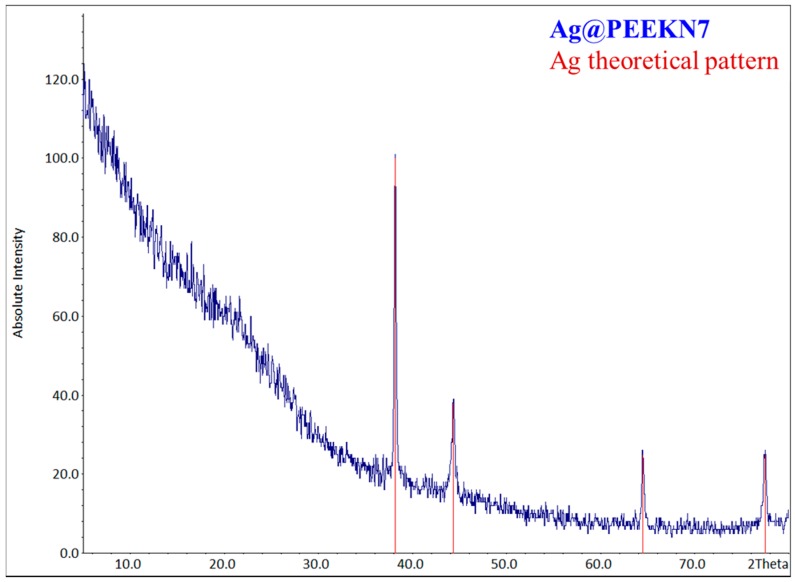
X-ray powder diffractogram of the precipitate of silver nitrate with **PEEKN7** after TGA analysis (blue) and theoretical silver pattern (red).

In order to combine silver compounds with our polymers, two different methods were applied. As direct complexation of **PEEKN5** with silver salts was not possible due to the insolubility in many solvents, we first complexed the soluble polymer **PEEKN7** with silver salts and tried to obtain a silver coordination polymer type material. Thus, mixing a solution of silver nitrate in methanol and a solution of **PEEKN7** in THF in a ratio 1:2 gave instantly an amorphous precipitate. TGA analysis (Figure 12) of this precipitate showed two mass losses, one starting at 150 °C and finishing at 270 °C, which could correspond to trapped solvent due to the instantaneous precipitation of the polymer. The second mass loss started at 420 °C, the temperature at which the polymer also starts to degrade. X-ray analysis of the TGA residue (Figure 13) confirms the existence of metallic silver. Indeed, silver nitrate decomposes at 450 °C to yield metallic silver [34].

**Figure 14:**
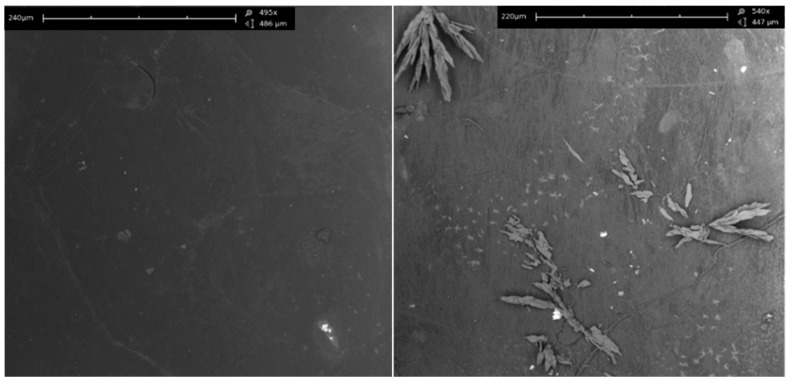
SEM pictures of **PEEKN5** alone (**left**); with silver complex 24 h (**right**).

We then applied on the surface of the insoluble **PEEKN5**, a silver coating in order to prove the ability of the pyridine group in PEEKNx to bind silver ions. Pieces of **PEEKN5** (total surface: 1 cm^2^) were immersed in 10 mL of an aqueous solution of silver nitrate for 24 h without any agitation. The remaining solution was subjected to an ICP-OES measurement and we always observed an uptake of 10% of the initial amount of silver ions. In a next procedure, we coated similar pieces of **PEEKN5** with the silver coordination polymer **A** (**A** = [Ag(L)NO_3_]_n_, with L = ethanediyl bis(isonicotinate) described previously [11,15], and from which we know that it has antimicrobial properties. Crystals of the silver complex **A** of different sizes from 5 to 100 μm, well dispersed on the surface, were observed by SEM pictures (Figure 14). XRPD diffractograms (data not shown) confirmed the presence of the silver complex and similar coating structures as for the metallic surfaces were obtained [11,15,16].

### 3.4. Antimicrobial Properties of **PEEK** Derivatives

To assess the antimicrobial properties of the silver coated **PEEKN5** derivatives, the well-known Kirby-Bauer assays were performed as silver compounds were shown to be active against both Gram-positive and Gram-negative bacteria [11,15,16,17]. The size of the inhibition zone is usually related to the level of antimicrobial activity present in the sample or product. A larger zone of inhibition usually means that the antimicrobial compound is more potent. The silver coated **PEEKN5** derivative showed a good antimicrobial activity against *E. coli* strains at a bacterial loading of 10^6^ CFU·mL^−1^ with an inhibition zone of 17.5 mm in diameter. Different amounts of silver nitrate (9.75, 40.45, 161.81, and 647.22 µg·mL^−1^) impregnated on Watmann paper were also tested against *E. coli* with inhibition zones ranging from 11 to 15 mm in diameter. Compared to the previous studies on silver compounds [11,15,17], in which the inhibition zone ranged from 15 to 20 mm and from above statement on silver ions, the silver coated **PEEKN5** compounds are active against Gram-positive bacteria and could also act against Gram-negative strains.

## 4. Conclusions

We have successfully synthetized a new type of polyether ether ketone with a pyridine side group in order to bind silver ions to the polymer. Different derivatives of hydroquinone were used in order to lower the melting point of the polymer. Crystal structures of the monomers with different silver salts were obtained with a linear coordination between two pyridine groups and the silver ions in all cases. Depending of the solubility of the PEEK derivatives, direct silver mixing and silver coating were applied in order to obtained silver coordination based material for antimicrobial purposes. Incorporation of silver ions into the polymer was attested by TGA and XRPD measurements. **PEEKN5** derivative was coated with the well-known silver coordination compound known to be active against bacteria. We showed that silver-coated **PEEKN5** had good antimicrobial properties against *E. coli* strains and may act also against other Gram-negative and Gram-positive bacteria. This new silver based **PEEK** derivative are ideal candidates for such antimicrobial coatings.

## Figures and Tables

**Table 1:** Glass transition, melting and decomposition temperature of the synthetized polymers. **PEEK** values are given for comparison.

PEEKN Derivative	Glass Transition Temperature °C	Melting Temperature °C	Decomposition Temperature °C
**PEEK** [4]	140	340	>400
**PEEKN5**	190	230	380
**PEEKN7**	165	270	380

## Supplementary Materials

Supplementary Materials can be found at www.mdpi.com/2073-4360/8/6/208/s1.

## Abbreviations

The following abbreviations are used in this manuscript:

PEEK	Polyether Ether Ketone
THF	Tetrahydrofuran
EtOH	Ethanol
NMR	Nuclear Magnetic Resonance
IR	Infra-red
MS	Mass Spectroscopy
TGA	Thermal Gravimetric Analysis
DSC	Differential Scanning Calorimetry
XRPD	X-ray Powder Diffractogram
DMTA	Dynamic Mechanical Thermal Analysis
ICP	Induced Coupled Plasma
SEM	Scanning Electron Microscopy
Tg	Glass Transition Temperature
Tm	Melting Temperature

## References

[1] Wei J., Lu R., Liu F. (2010). Novel, highly efficient polymeric benzophenone photoinitiator containing coinitiator moieties for photopolymerization. Polym. Adv. Technol..

[2] Papilloud S., Baudraz D. (2002). Analysis of food packaging UV inks for chemicals with potential to migrate into food simulants. Food Addit. Contam..

[3] Staniland P.A. (1986). Thermoplastische aromatische polyetherketone.

[4] Van der Vegt A., Govaert L. (2003). Plymeren van Keten tot Kunststof.

[5] Seferis J.C. (1986). Polyetheretherketone (PEEK): Processing-structure and properties studies for a matrix in high performance composites. Polym. Compos..

[6] Corvelli A.A., Roberts J.C., Biermann P.J., Cranmer J.H. (1999). Characterization of a peek composite segmental bone replacement implant. J. Mater. Sci..

[7] Mano J.F., Sousa R.A., Boesel L.F., Neves N.M., Reis R.L. (2004). Bioinert, biodegradable and injectable polymeric matrix composites for hard tissue replacement: State of the art and recent developments. Compos. Sci. Technol..

[8] Darouiche R.O. (2004). Treatment of infections associated with surgical implants. N. Engl. J. Med..

[9] Barton A.J., Sagers R.D., Pitt W.G. (1996). Bacterial adhesion to orthopedic implant polymers. J. Biomed. Mater. Res..

[10] Vaudaux P., François P., Berger-Bächi B., Lew D.P. (2001). *In vivo* emergence of subpopulations expressing teicoplanin or vancomycin resistance phenotypes in a glycopeptide-susceptible, methicillin-resistant strain of *Staphylococcus aureus*. J. Antimicrob. Chemother..

[11] Gordon O., Vig Slenters T., Brunetto P.S., Villaruz A.E., Sturdevant D.E., Otto M., Landmann R., Fromm K.M. (2010). Silver coordination polymers for prevention of implant infection: Thiol interaction, impact on respiratory chain enzymes, and hydroxyl radical induction. Antimicrob. Agents Chemother..

[12] Vig Slenters T., Sague J.L., Brunetto P.S., Zuber S., Fleury A., Mirolo L., Robin A.Y., Meuwly M., Gordon O., Landmann R. (2010). Of chains and rings: Synthetic strategies and theoretical investigations for tuning the structure of silver coordination compounds and their applications. Materials.

[13] Fromm K.M. (2011). Give silver a shine. Nat. Chem..

[14] Eckhardt S., Brunetto P.S., Gagnon J., Priebe M., Giese B., Fromm K.M. (2013). Nanobio silver: Its interactions with peptides and bacteria, and its uses in medicine. Chem. Rev..

[15] Brunetto P.S., Vig Slenters T., Fromm K.M. (2011). *In vitro* biocompatibility of new silver(I) coordination compound coated-surfaces for dental implant applications. Materials.

[16] Fromm K.M. (2013). Silver coordination compounds with antimicrobial properties. Appl. Organomet. Chem..

[17] Varisco M., Khanna N., Brunetto P.S., Fromm K.M. (2014). New antimicrobial and biocompatible implant coating with synergic Silver-Vancomycin conjugate action. ChemMedChem.

[18] Girard J., Brunetto P.S., Braissant O., Rajacic Z., Khanna N., Landmann R., Daniels A.U., Fromm K.M. (2013). Development of a polystyrene sulfonate/silver nanocomposite with self-healing properties for biomaterial applications. Comptes Rendus Chim..

[19] Kamm O., Mattheww A.O. (1922). *p*-Nitrobenzoic acid. Org. Synth..

[20] Weikert R.J., Bingham S., Emanuel M.A., Fraser Smith E.B., Loughhead D.G., Nelson P.H., Poulton A.L. (1991). Synthesis and anthelmintic activity of 3′-benzoylurea derivatives of 6-phenyl-2,3,5,6-tetrahydroimidazo[2,1-b]t hiazole+. J. Med. Chem..

[21] Gors H.G., Horner P.J., Jansons V. (1989). Friedel-crafts preparation of aromatic ketones. U.S. Patent.

[22] Cosier J., Glazer A.M. (1986). A nitrogen-gas-stream cryostat for general X-ray diffraction studies. J. Appl. Cryst..

[23] Blanc E., Schwarzenbach D., Flack H.D. (1991). The evaluation of transmission factors and their first derivatives with respect to crystal shape parameters. J. Appl. Cryst..

[24] Burla M.C., Caliandro R., Camalli M., Carrozzini B., Cascarano G.L., de Caro L., Giacovazzo C., Polidori G., Spagna R. (2005). SIR2004: An improved tool for crystal structure determination and refinement. J. Appl. Cryst..

[25] Sheldrick G.M. (1997). SHELX-97. Program for Crystal Structure Refinement.

[26] Steiner T. (2002). The hydrogen bond in the solid state. Angew. Chem. Int. Ed..

[27] Etter M.C. (1990). Encoding and decoding hydrogen-bond patterns of organic compounds. Acc. Chem. Res..

[28] Hoffmann R., Swenson J.R. (1970). Ground- and excited-state geometries of benzophenone. J. Phys. Chem..

[29] Maginn S.J., Davey R.J. (1994). 4,4′-difluorobenzophenone. Acta Crystallogr. Sect. C.

[30] Wu L.-L., Yang C.-L., Lo F.-C., Chiang C.-H., Chang C.-W., Ng K.Y., Chou H.-H., Hung H.-Y., Chan S.I., Yu S.S.-F. (2011). Tuning the regio- and stereoselectivity of C–H activation in n-octanes by cytochrome P450 BM-3 with fluorine substituents: Evidence for interactions between a C–F bond and aromatic π systems. Chem. Eur. J..

[31] Laia Y.H., Kuoa M.C., Huanga J.C., Chena M. (2007). On the PEEK composites reinforced by surface-modified nano-silica. Mater. Sci. Eng. A.

[32] Johansson M., Malmström E., Hult A. (1993). Synthesis, characterization, and curing of hyperbranched allyl ether-maleate functional ester resins. J. Polym. Sci. A.

[33] Díez-Pascual A.M., Naffakh M., Gómez-Fatou M.A. (2011). Mechanical and electrical properties of novel poly(ether ether ketone)/carbon nanotube/inorganic fullerene-like WS_2_ hybrid nanocomposites: experimental measurements and theoretical predictions. Mater. Chem. Phys..

[34] Paulik F., Paulik J., Arnold M. (1985). Examination of the decomposition of AgNO_3_ by means of simultaneous EGA and TG method under conventional and quasi isothermal circumstances. Thermochim. Acta.

