# Supplementary material for: Synthesis of New Polyether Ether Ketone Derivatives with Silver Binding Site and Coordination Compounds of Their Monomers with Different Silver Salts
Source: Polymers (Basel). 2016 May 30;8(6):208. doi: 10.3390/polym8060208 (PMC6432468; doi:10.3390/polym8060208)
Supplement: Supplementary file 1 [file polymers-08-00208-s001.pdf]

Supplementary Materials: Synthesis of New Polyether Ether Ketone Derivatives with Silver Binding Site and Coordination Compounds of Their Monomers with Different Silver Salts

Jérôme Girard, Nathalie Joset, Aurélien Crochet, Milène Tan, Anja Holzheu, Priscilla S. Brunetto and Katharina M. Fromm

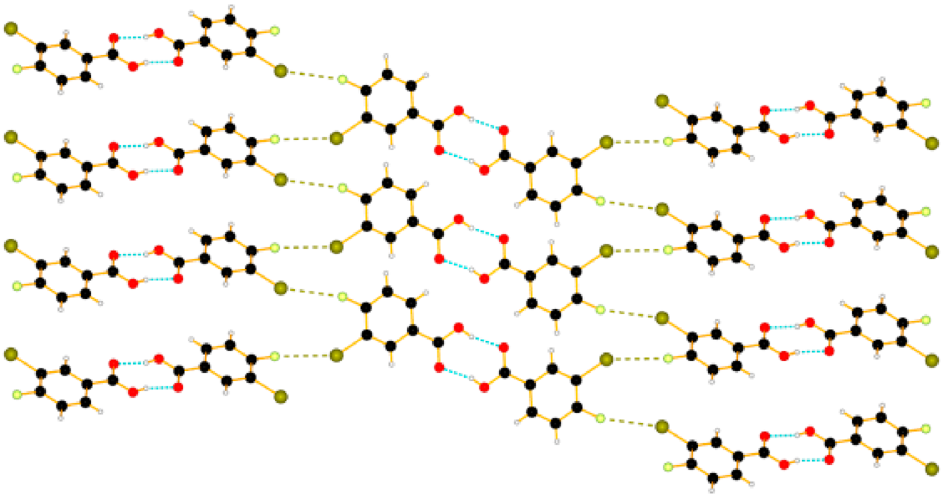

Figure S1. Packing structure of compound **2**.

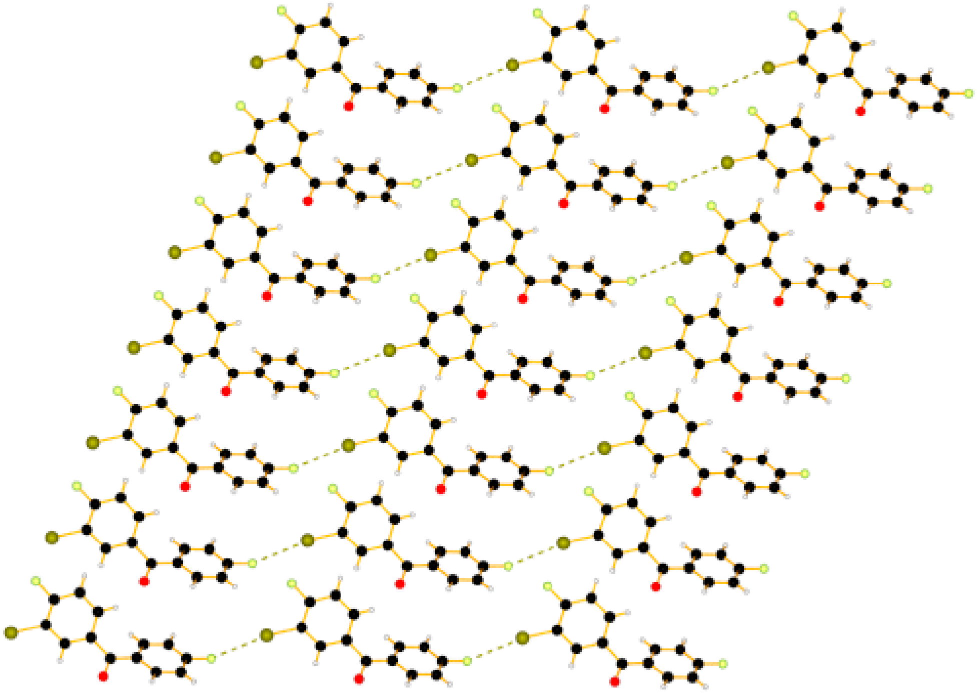

Figure S2. Packing structure of compound **3**.

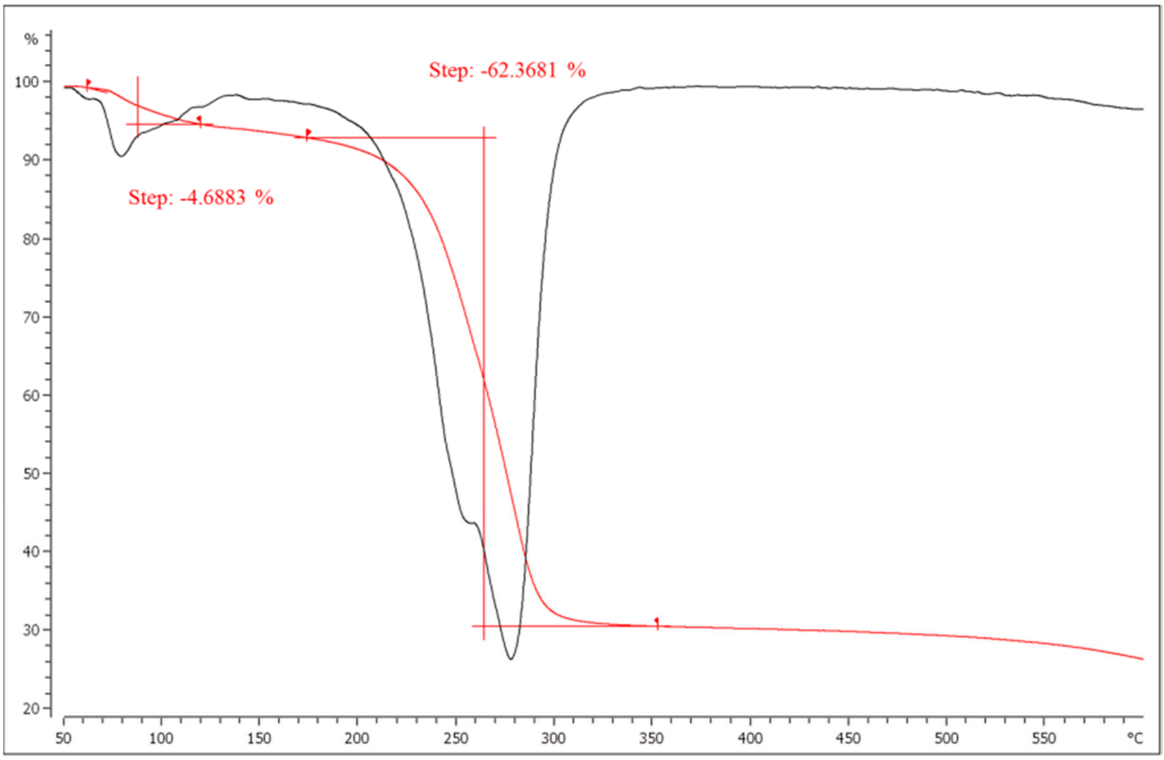

Figure S3. TGA of compound **4b**.

Table S1. Crystal data and structures refinement for X-ray structures.

**Compound**	**2**	**3**	**4**	**4a**	**4b**	**5a**
CCDC number	CCDC-1411840	CCDC-1411841	CCDC-1411842	CCDC-1411843	CCDC-1411844	CCDC-1411845
Empirical formula	$C_7H_4BrFO_2$	$C_{13}H_7BrF_2O$	$C_{18}H_{11}F_2NO$	$C_{37}H_{18}AgF_7N_2O_5S$	$C_{37}H_{25}AgF_4N_2O_3$	$C_{37}H_{26}AgF_4N_3O_6$
Formula weight (g·mol^{-1})	219.01	297.10	295.28	843.47	729.46	792.48
Temperature	200(2) K	200(2) K	200(2) K	200(2) K	200(2) K	200(2) K
Wavelength	0.71073 Å	1.54186 Å	0.71073 Å	0.71073 Å	1.54186 Å	0.71073 Å
Crystal system	Monoclinic	Monoclinic	Monoclinic	Monoclinic	Monoclinic	Monoclinic
Space group	$P\ 2_1/n$	$P2_1/c$	$P\ 2_1/c$	$C\ 2/c$	$P\ 2_1/c$	$P\ 2_1/n$
Unit cell dimensions	a = 4.6710(10) Å<br>b = 5.8829(13) Å<br>c = 26.760(5) Å<br>β = 93.320(17)°	a = 6.2091(8) Å<br>b = 14.4523(13) Å<br>c = 12.2712(13) Å<br>β = 93.510(10)°	a = 14.5724(8) Å<br>b = 12.5173(14) Å<br>c = 7.5171(15) Å<br>β = 101.958(8)°	a = 38.964(3) Å<br>b = 7.9942(5) Å<br>c = 23.6965(14) Å<br>β = 113.452(4)°	a = 24.301(2) Å<br>b = 3.8259(3) Å<br>c = 34.880(3) Å<br>β = 92.941(7)°	a = 7.5262(4) Å<br>b = 12.1802(4) Å<br>c = 35.5300(17) Å<br>β = 96.000(4)°
Volume	734.1(3) Å^3	1,099.1(2) Å^3	1,341.4(3) Å^3	6,771.3(7) Å^3	3,238.6(5) Å^3	3,239.2(3) Å^3
Z	4	4	4	8	4	4
Density (calculated) (Mg/m^3)	1.982	1.795	1.462	1.655	1.496	1.625
F(000)	424	584	608	3,360	1,472	1,600
Crystal size (mm^{3})	0.2 × 0.07 × 0.05	0.25 × 0.05 × 0.03	0.180 × 0.120 × 0.050	0.220 × 0.10 × 0.030	0.36 × 0.04 × 0.02	0.250 × 0.117 × 0.040
Theta range for data collection	3.05° to 25.00°	4.73° to 62.72°	1.43° to 25.00°	1.76° to 25.00°	1.82° to 55.00°	1.77° to 25.00°
Index ranges	$-5 \le h \le 5$<br>$-6 \le k \le 6$<br>$-31 \le l \le 31$	$-6 \le h \le 6$<br>$0 \le k \le 16$<br>$0 \le l \le 14$	$-17 \le h \le 17$<br>$-14 \le k \le 14$<br>$-8 \le l \le 8$	$-46 \le h \le 46$<br>$-9 \le k \le 9$<br>$-27 \le l \le 28$	$-22 \le h \le 25$<br>$-3 \le k \le 4$<br>$-37 \le l \le 37$	$-8 \le h \le 8$<br>$-14 \le k \le 14$<br>$-42 \le l \le 42$
Reflections collected	7,325	1,563	16,600	63,084	20,891	38,973
Independent reflections	1,271 [R(int) = 0.1760]	1,563 [R(int) = 0.000]	2,346 [R(int) = 0.1828]	5,917 [R(int) = 0.1197]	4,025 [R(int) = 0.0952]	5,551 [R(int) = 0.0775]
Absorption correction	Empirical	Integration	None	None	Integration	Integration
Max. and min. transmission	0.7245 and 0.2754	0.9943 and 0.8995	/	/	0.7674 and 0.2321	0.9420 and 0.8631
Refinement method	Full-matrix least-squares on F^2	Full-matrix least-squares on F^2	Full-matrix least-squares on F^2	Full-matrix least-squares on F^2	Full-matrix least-squares on F^2	Full-matrix least-squares on F^2
Data/restraints/parameters	1,271/0/101	1,563/0/154	2,346/0/199	5,917/0/505	4,025/0/425	5,551/0/443
Goodness-of-fit on F^2	1.140	0.990	1.021	1.037	0.981	1.055
Final R indices [I > 2σ(I)]	$R1$ = 0.0755<br>$wR2$ = 0.1412	$R1$ = 0.0464<br>$wR2$ = 0.1118	$R1$ = 0.0700<br>$wR2$ = 0.1392	$R1$ = 0.0711<br>$wR2$ = 0.1836	$R1$ = 0.0778<br>$wR2$ = 0.2226	$R1$ = 0.0987<br>$wR2$ = 0.2496
R indices (all data)	$R1$ = 0.0898<br>$wR2$ = 0.1478	$R1$ = 0.0799<br>$wR2$ = 0.1274	$R1$ = 0.1168<br>$wR2$ = 0.1565	$R1$ = 0.0955<br>$wR2$ = 0.1981	$R1$ = 0.0944<br>$wR2$ = 0.2399	$R1$ = 0.1394<br>$wR2$ = 0.2752
Largest diff. peak and hole	0.859 and −0.537 e·Å^{-3}	0.506 and −0.531 e·Å^{-3}	0.191 and −0.205 e·Å^{-3}	1.409 and −1.211 e·Å^{-3}	2.482 and −1.217 e·Å^{-3}	0.804 and −1.017 e·Å^{-3}

© 2016 by the authors; licensee MDPI, Basel, Switzerland. This article is an open access article distributed under the terms and conditions of the Creative Commons Attribution (CC-BY) license (http://creativecommons.org/licenses/by/4.0/).
